# The Incidence of Contrast-Induced Nephropathy Following Computed Tomography and Associated Risk Factors

**DOI:** 10.1155/rrp/7484380

**Published:** 2025-07-11

**Authors:** Reza Mosaddegh, Fatemeh Mohammadi, Aydin Mohammad Valipour, Seyed Mohammad Hosseini, Mobin Naghshbandi, Mobina Yarahmadi, Nazanin Alaei Faradonbeh

**Affiliations:** ^1^Emergency Medicine Management Research Center, Health Management Research Institute, School of Medicine, Iran University of Medical Sciences, Tehran, Iran; ^2^Student Research Committee, School of Medicine, Iran University of Medical Sciences, Tehran, Iran; ^3^Physiology Research Center, Iran University of Medical Sciences, Tehran, Iran

**Keywords:** acute kidney injury, contrast-induced nephropathy, CT-scan

## Abstract

**Introduction:** Contrast-induced nephropathy (CIN) is a common adverse effect of imaging using intravenous contrast media. In this study, we aimed to retrospectively investigate the incidence of CIN following contrast media administration and determine the significant risk factor.

**Methods:** This study is a retrospective cross-sectional study conducted in Firoozgar Hospital in Tehran during 2019 to 2020. A total of 160 patients who underwent computed tomography (CT-scan) with IV contrast in the mentioned time period were enrolled in the study. The main dependent variable was serum creatinine level after exposure to contrast which was measured in the following 48–96 h after imaging. Independent variables such as patients' demographics (age and sex), patients' comorbidities (diabetes, hypertension, coronary artery disease, heart failure, peripheral vascular disease, liver failure, and anemia), periprocedural details (hydration procedure, hemoglobin level, hematocrit, serum creatinine level, and glomerular filtration rate prior to exposure to contrast), and patients' drug history were collected by reviewing their medical reports.

**Results:** A total of 14 patients (8.8%) developed CIN, forming the CIN-positive group. The remaining patients, who did not develop CIN, were categorized as the CIN-negative group. There was no statistically significant difference between the two groups in terms of age or gender.

**Conclusion:** 8.8% of patients developed CIN following contrast administration. Among risk factors, the only effectual risk factor was the initial serum creatinine level.

## 1. Introduction

Nowadays, radiologic contrast media (RCM) are being widely used alongside computed tomography (CT-scan) to evaluate pathologies and determine therapeutic responses. Despite the fact that RCM are proven as useful tools for diagnostic and therapeutic methods, they are attributed to numerous adverse effects, of which hypersensitivity reactions and contrast-induced nephropathy (CIN) are the most common [1].

CIN is defined as the development of acute kidney failure (AKI) within 48 h of intravenous (IV) contrast administration after excluding other possible nephrotoxic mechanisms. Two common definitions are proposed for CIN including the Kidney Disease Improving Global Outcomes (KDIGO) and the Acute Kidney Injury Network (AKIN) criteria. The KDIGO and AKIN criteria define CIN as an absolute serum creatinine increase of ≥ 0.3 mg/dL above baseline within 48 h or a percentage increase in serum creatinine of ≥ 50% above baseline within 7 days or 48 h, respectively [2, 3].

Previous reports indicate that CIN occurs in 4% to 20% of patients after intra-arterial administration of contrast material in coronary angiography and is the third cause of nosocomial acute renal failure [4–7]. Investigators have indicated many risk factors for CIN including chronic kidney disease (CKD), advanced age, diabetes mellitus, congestive heart failure (CHF), hypovolemia, and high contrast volume [8–10]; nevertheless, the exact mechanism of the induced nephropathy has not hitherto been well elucidated. Two theories are proposed as the possible mechanisms of CIN: renal vasoconstriction (probably due to changes in nitric oxide, endothelin, and adenosine) and direct tubular damage. Other possible mechanisms are activation of tubuloglomerular feedback, regional hypoxia, and production of reactive oxygen species (ROS). However, no exact relationship between the rate of contraction of renal vessels and the rate of subsequent increase in creatinine has been observed, and the occurrence of kidney failure is mostly attributed to the decrease in blood flow in the renal cortex [11, 12].

According to the increasing rate of CIN and the importance of its prevention, in this article, we retrospectively investigated the incidence of CIN following contrast media administration in Firoozgar Center in Tehran, Iran, with the aim to discover modifiable risk factors to estimate the development of CIN in order to prevent it in our hospital.

## 2. Methods

### 2.1. Study Population and Eligibility Criteria

This study is a retrospective cross-sectional study conducted in Firoozgar Hospital in Tehran during 2019 to 2020. A total of 160 patients who underwent CT-scan with IV contrast in the mentioned time period were enrolled in the study. Inclusion criteria comprised of patients who had undergone IV contrast CT-scan, patients with recorded creatinine level 48 to 96 h prior to the imaging, and patients who had not taken nephrotoxic medications. Exclusion criteria consisted of patients who were hemodynamically unstable, patients in need of nephrotoxic medications, and patients with more than 20% missed data in their medical records.

### 2.2. CIN Definition and Variables

In our study, CIN was defined as either a relative increase in serum creatinine of ≥ 25% compared to the baseline creatinine value within 48 to 96 hours after exposure to contrast media, or an absolute increase in serum creatinine of ≥ 0.5 mg/dL within the same time frame. The main dependent variable was serum creatinine level after exposure to contrast which was measured in the following 48–96 h after imaging. Independent variables such as patients' demographics (age and sex), patients' comorbidities (diabetes, hypertension, coronary artery disease (CAD), heart failure, peripheral vascular disease, liver failure, and anemia), periprocedural details (hydration procedure, hemoglobin (Hb) level, hematocrit (Hct), serum Cr level, and glomerular filtration rate (GFR) prior to exposure to contrast), and patients' drug history were collected by reviewing their medical reports. All patients were hydrated prior injection with the same procedure.

### 2.3. Contrast Media and Imaging Protocol

All patients received the same nonionic, low-osmolar iodinated contrast agent (iohexol, GE Healthcare) at a dose of 1.5 mL/kg, with a typical volume ranging from 80 to 120 mL. The contrast was administered intravenously using a power injector. The indications for CT scans were as follows: thoracic CT (*n* = 60, 37.5%), abdominal/pelvic CT (*n* = 70, 43.8%), cranial CT (*n* = 20, 12.5%), and other regions (*n* = 10, 6.2%). All examinations were performed on a Siemens SOMATOM Definition AS+ 128-slice CT scanner.

### 2.4. Hydration Protocol

In our hospital, it is standard procedure for all patients scheduled for elective contrast-enhanced CT scans to receive 500 mL isotonic saline intravenously over 1 h prior to contrast administration. This prehydration protocol is routinely implemented for elective and inpatient cases. Patients who underwent emergency CT scans without prehydration were excluded from this study.

### 2.5. Statistical Analysis

The data analysis was done using SPSS Version 20.0. We expressed data using mean ± standard deviation (SD) for continuous variables and counts and percentages for categorical variables. Also, we used independent T-test to compare continuous variables and chi-square test to compare categorical variables. To determine risk factors of CIN, logistic regression analysis was utilized. A *p* value < 0.05 was considered statistically significant.

### 2.6. Ethical Approval

Ethical approval for this study was obtained from the Ethics Committee of Iran University of Medical Sciences (Approval No. IR.IUMS.FMD.REC.1394.861011063).

## 3. Results

**Figure 1:**
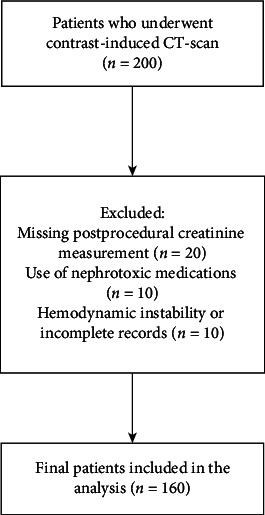
Flowchart of the subject recruitment.

A total of 160 subjects were included in this study of which 73 were females (45.6%) and 87 were males (54.4%). During the study period, 200 subjects underwent contrast-enhanced CT scans. Of these, 20 were excluded due to missing postprocedural creatinine measurements, 10 due to use of nephrotoxic medications, and 10 due to incomplete medical records or hemodynamic instability. Therefore, a total of 160 subjects were included in the final analysis. A flowchart of the subject recruitment is depicted in Figure 1. The age of the subjects ranged from 17 to 87 years old (mean age 52.4 ± 17.5). Our subjects had a mean Hb level of 11.1 ± 2.3 mg/dL, Hct level of 33.6 ± 1.2%, and mean GFR of 84.6 ± 27.2 mL/min/1.73 m^2^. The median serum Cr level prior and after CT-scan was 0.92 ± 0.24 mg/dL and 0.95 ± 0.68 mg/dL, respectively. In addition, 20.6% of all subjects had hypertension, 13.8% had liver failure, 11.9% had anemia, 9.4% had DM, 8.1% had CAD, 7.5% had heart failure, and 1.3% had peripheral vascular disease.

A total of 14 subjects (8.8%) developed CIN and were classified as the CIN (+) group. The remaining subjects without CIN comprised the CIN (−) group. There was no statistically significant difference between the two groups in terms of age or gender. Furthermore, no significant difference was observed between CIN (+) and CIN (−) groups regarding the frequency of comorbidities: hypertension, diabetes, CAD, heart failure, peripheral vascular disease, liver failure, and anemia (Table 1).

The mean Hb levels, mean Hct levels, and GFR before the procedure were almost similar in both groups, and no statistically significant difference was found. On the other hand, the mean creatinine level before the CT-scan in the CIN (+) group was 0.72 ± 0.22 and that in the CIN (−) group was 0.94 ± 0.23, which had a significant statistical difference (*p* value = 0.001) (Table 2).

Logistic regression was performed to identify independent predictors of CIN. The following variables were included in the model: age, gender, diabetes mellitus, hypertension, CAD, heart failure, peripheral vascular disease, liver failure, anemia, preprocedural serum creatinine, Hb, Hct, and GFR. Among these, only preprocedural serum creatinine was independently associated with CIN (odds ratio (OR): 2.87, 95% confidence interval (CI): 1.45–5.67, *p*=0.002). Other variables were not statistically significant: age (OR: 1.01, 95% CI: 0.97–1.05, *p*=0.62), diabetes mellitus (OR: 1.32, 95% CI: 0.37–4.67, *p*=0.67), hypertension (OR: 1.48, 95% CI: 0.48–4.61, *p*=0.50), and male gender (OR: 0.94, 95% CI: 0.28–3.10, *p*=0.92). A summary of the logistic regression is provided in Table 3.

## 4. Discussion

CIN is defined as the sudden decrease in the renal function following IV contrast media injection. Several previous studies have reported different numbers regarding the incidence of CIN following contrast injection. Turedi et al. [13] reported an incidence of 23.7%, while studies by Dagar et al. [14] and McGillicuddy et al. [15] reported CIN incidence as 4.9% and 1.9%, respectively. The incidence of CIN in our sample was 8.8%. This rate is within the expected range reported in other articles. Because of diversity in risk factors, the population studied, and the definition of CIN, the rate has been reported as 3% to 20%. Moreover, based on the type of the procedure, the rate can be different. For instance, the rate of CIN after carotid artery stenting is 21% [16, 17].

Numerous previously published studies have demonstrated that CIN can result in long-term mortality and cardiovascular adverse events [18, 19], even in subjects who are in a stable hemodynamic status [20]. For instance, in a study conducted by Mitchell et al. [21] in 2015 on 633 subjects who underwent CT scan with contrast material in the emergency department, the incidence of CIN was 11% (70 of 633). 15 subjects (2%) died within 45 days, 6 of whom had CIN. 7 (1%). Among 6 subjects with CIN and severe renal failure, 4 died subsequently. In this study, CIN was shown to be associated with an increased risk of acute renal failure and mortality from renal failure.

According to previous studies, some risk factors like age, comorbidities, and preprocedural hydration can influence the rate of CIN. The current study contributes to the ongoing debate regarding risk factors for CIN, specifically in the emergency setting. Interestingly, this study found that the mean baseline serum creatinine was significantly lower in the CIN (+) group compared to the CIN (−) group (0.72 ± 0.22 vs. 0.94 ± 0.23, *p*=0.001). This finding contrasts with many previous studies, which report higher baseline creatinine as a risk factor for CIN development. Interestingly, in contrast to prior studies, our results showed that patients who developed CIN had lower baseline creatinine levels than those who did not. This paradoxical result may be due to selection bias, early-stage renal stress without overt dysfunction, or a reflection of the dynamic physiological changes in acute care settings. It also emphasizes the need to consider additional biomarkers or patient-specific risk stratification models rather than relying solely on baseline creatinine [22, 23]. For instance, in a retrospective study conducted by Gorelik et al., the researchers investigated the safety of contrast media–enhanced CT-scan in subjects with advanced renal dysfunction. The subjects were divided into two groups of subjects with GFR > 30 and GFR < 30, respectively. The results indicated that subjects with low GFR had an increased risk to develop postcontrast acute kidney injury (PC-AKI) than other subjects. Furthermore, regression analysis of all subjects undergoing CT-scan with a GFR < 30 demonstrated that contrast enhancement increased the risk of PC-AKI by 51% [24].

A recent meta-analysis of 21 cohort studies, encompassing data from over 169,000 patients, demonstrated no statistically significant difference in the overall risk of AKI between individuals undergoing contrast-enhanced CT and those receiving unenhanced CT (OR: 0.97; 95% CI: 0.85–1.11; *p*=0.64), irrespective of baseline renal function or underlying comorbidities. However, the study identified hypertension and an estimated glomerular filtration rate (eGFR) ≤ 30 mL/min/1.73 m^2^ as independent predictors of PC-AKI [25].

In comparison, the present study reported a CIN incidence of 8.8%, with baseline serum creatinine level emerging as the only significant predictor of CIN development. Contrary to the meta-analysis findings, no statistically significant associations were found between CIN and common comorbid conditions such as diabetes mellitus, hypertension, CKD, CHF, liver disease, or anemia. These discrepancies may be attributed to variations in study population characteristics, sample size, and the lack of laboratory stratification (e.g., HbA1c levels in diabetic patients or eGFR staging for renal function).

These contrasting outcomes underscore the complex and multifactorial etiology of CIN and support the growing consensus that the nephrotoxic potential of contrast media is likely overestimated in the general population. Nonetheless, careful risk stratification remains essential, particularly for patients with significantly impaired renal function or uncontrolled hypertension.

In contrast with our results, in the study conducted by Sonhaye et al., the researchers investigated the rate of CIN development after the IV contrast-enhanced CT-scan procedure. Their study strata comprised of a total of 620 subjects undergoing CT-scan in the emergency room using IV contrast and 672 subjects taking CT-scan without IV contrast. Among the subjects receiving IV contrast, only 3% percent developed CIN. Moreover, no subject had renal impairment upon discharge. The results of the multivariate analysis showed no increased risk of AKI related to IV contrast [26].

Contrary to many previous studies, we did not find a statistically significant relationship between common comorbidities (e.g., diabetes, hypertension, and CKD) and CIN in our sample. This may be due to heterogeneity in disease severity or incomplete lab markers like HbA1c, which future studies should include for more nuanced interpretation [17, 18, 27, 28].

In addition to what was mentioned, previous experiments have discovered that advanced age [29, 30] and sex [20, 29] can act as predictors for CIN. Nevertheless, in this study and some other studies [23], age was not a predictor for CIN. Although it is clear that the incidence of comorbidities increases as patients get older, this factor should be considered when interpreting the results [28]. Kaya et al. demonstrated that the volume of contrast material had a significant effect on incidence of CIN in their survey [29]. Hb level and GFR were reported to act as a predictor for CIN [28, 31, 32]. A study conducted on 250 patients with baseline eGFR ≥ 45 mL/min/1.73 m^2^ found a 10% incidence of CIN, defined by a 25% increase or an absolute rise of 0.5 mg/dL in serum creatinine postprocedure. The majority of CIN cases were transient, although a small number of patients developed renal failure or died. Importantly, dehydration, preexisting renal disease, cardiac failure, prior contrast exposure, and the volume of contrast administered showed statistically significant correlations with CIN development (*p* < 0.05). Conversely, demographic factors, baseline serum creatinine or eGFR, diabetes mellitus, hypertension, nephrotoxic medication use, routine hematologic abnormalities, and contrast media characteristics did not significantly influence CIN risk [33].

Acknowledging the risk factors of CIN can assist radiologists to select appropriate CT protocol [34].

One common and universally acceptable strategy to prevent CIN is hydrating the patients [20, 35]. In this way, volume expansion suppresses the renin-angiotensin-aldosterone system, increases the excretion of contrast material, reduces tubuloglomerular feedback, and decreases both the direct renal toxicity of the contrast agent and the release of vasoconstrictors [36]. Furthermore, some experiments proved that using sodium chloride regimen for 12 h before exposure to material was more efficient than 1 h sodium bicarbonate preexposure regimen [37]. However, it should be considered that hydration can cause pulmonary edema in CHF patients, so usually these patients do not receive adequate hydration and they are more susceptible to CIN. Some investigators demonstrated that it is not necessary to use intravascular fluids, in order to hydration, for all outpatients and it can be reserved for hospitalized population or high-risk outpatients [38]. Many studies declared that administrating of N-acetylcysteine can be useful to prevent CIN [39–41] but Traub et al. reported that N-acetylcysteine had no benefit in lowering the risk of CIN after emergency enhanced CT [42].

Likewise, it is accepted that nitrate administration, when combined with hydration, can reduce the incidence of CIN [35]. Previous studies hypothesized that nitrates can protect medulla against hypoxia and oxidative stress [43–45]. Another way to prevent CIN, particularly in patients with ST elevation myocardial infarction, is taking statins [46] especially pravastatin [47] and rosuvastatin [48].

## 5. Conclusion

In conclusion, the results showed that the incidence of CIN in the patients of this study was 8.8%. Among the risk factors, only the level of creatinine before the CT scan was significantly related to the incidence of contrast nephropathy. No significant relationship was found between comorbidity and incidence of contrast nephropathy. In order to achieve a more expanded understanding regarding this matter, this study suggests conducting more extensive studies in this field with a larger sample size in the future.

## 6. Limitation

This study has some limitations. First, it is a single-institution survey and based on a small number of patients. Second, this is a retrospective study and we reviewed medical records; some of the records had impaired information. Finally, we could not follow patients to find out long-term effect of contrast materials.

## Figures and Tables

**Table 1:** Demographic data and comorbidities of the subjects.

Variable	Total *n* = 160	CIN (+) *n* = 14	CIN (−) *n* = 146	*p* value
Age (years)	17.5 ± 52.4	17.1 ± 54.4	52.3 ± 17.5	0.66
Gender, female (*n*, %)	73 (45.6%)	(43%)6	67 (46%)	0.82
Diabetes mellitus (*n*, %)	15 (9.4%)	2 (14.3%)	13 (9%)	0.5
Hypertension (*n*, %)	33 (20.6%)	4 (28.6%)	29 (20%)	0.4
Coronary artery disease (*n*, %)	13 (8.1%)	1 (7%)	12 (8.2%)	0.8
Heart failure (*n*, %)	12 (7.5%)	1 (7%)	11 (7.5%)	0.9
Peripheral vascular disease (*n*, %)	2 (1.3%)	0	2 (1.4%)	0.6
Liver failure (*n*, %)	22 (13.8%)	3 (21.4%)	19 (13%)	0.3
Anemia (*n*, %)	19 (11.9%)	0	19 (13%)	0.1

**Table 2:** Comparison of hematologic variables of subjects.

Variable	Total *n* = 160	CIN (+) *n* = 14	CIN (−) *n* = 146	*p* value
Hb (mg/dL)	11.1 ± 2.3	11.4 ± 1.1	11.1 ± 2.3	0.6
Hct (%)	33.6 ± 1.2	35.5 ± 2.6	37.7 ± 3.9	0.8
GFR (mL/min/1.73 m^2^)	84.6 ± 27.2	94 ± 31	83 ± 26	0.17
Primary Cr (mg/dL)	0.92 ± 0.24	0.72 ± 0.22	0.94 ± 0.23	0.001
Secondary Cr (mg/dL)	0.95 ± 0.68	1 ± 0.31	0.94 ± 0.71	0.47

**Table 3:** Logistic regression analysis for predictors of contrast-induced nephropathy.

Variable	Odds ratio (OR)	95% confidence interval	*p* value
Age (per year)	1.01	0.97–1.05	0.62
Male gender	0.94	0.28–3.10	0.92
Diabetes mellitus	1.32	0.37–4.67	0.67
Hypertension	1.48	0.48–4.61	0.50
Pre-CT serum creatinine	2.87	1.45–5.67	0.002^∗^

^∗^Significant.

## Data Availability

The data that support the findings of this study are available on request from the corresponding author. The data are not publicly available due to privacy or ethical restrictions.
